# Effect of single intralesional treatment of surgically induced equine superficial digital flexor tendon core lesions with adipose-derived mesenchymal stromal cells: a controlled experimental trial

**DOI:** 10.1186/s13287-017-0564-8

**Published:** 2017-06-05

**Authors:** Florian Geburek, Florian Roggel, Hans T. M. van Schie, Andreas Beineke, Roberto Estrada, Kathrin Weber, Maren Hellige, Karl Rohn, Michael Jagodzinski, Bastian Welke, Christof Hurschler, Sabine Conrad, Thomas Skutella, Chris van de Lest, René van Weeren, Peter M. Stadler

**Affiliations:** 1 Equine Clinic, University of Veterinary Medicine Hannover, Foundation, Bünteweg 9, 30559 Hannover, Germany; 2 Department of Equine Sciences, Faculty of Veterinary Medicine, Utrecht University, Yalelaan 112, 3584 CM Utrecht, The Netherlands; 3 Institute for Pathology, University of Veterinary Medicine Hannover, Foundation, Bünteweg 17, 30559 Hannover, Germany; 4 Pferdeklink Kirchheim, Nürtinger Straße 200, 73230 Kirchheim unter Teck, Germany; 5 Institute for Biometry, Epidemiology and Information Processing, University of Veterinary Medicine Hannover, Foundation, Bünteweg 2, 30559 Hannover, Germany; 6 Department of Orthopedic Trauma, Hannover Medical School, Carl-Neuberg-Straße 1, 30625 Hannover, Germany; 7 Laboratory for Biomechanics and Biomaterials, Department of Orthopaedic Surgery, Hannover Medical School, Anna-von-Borries-Straße 1-7, 30625 Hannover, Germany; 8 P.O. Box 1243, 72072 Tübingen, Germany; 9 Institute for Anatomy and Cell Biology, University of Heidelberg, Im Neuenheimer Feld 307, 69120 Heidelberg, Germany

**Keywords:** Horse, Ultrasonography, Ultrasound tissue characterization, Biomechanical testing, Histology, Biochemistry, MSC, mesenchymal stem cells, Tendon

## Abstract

**Background:**

Adipose tissue is a promising source of mesenchymal stromal cells (MSCs) for the treatment of tendon disease. The goal of this study was to assess the effect of a single intralesional implantation of adipose tissue-derived mesenchymal stromal cells (AT-MSCs) on artificial lesions in equine superficial digital flexor tendons (SDFTs).

**Methods:**

During this randomized, controlled, blinded experimental study, either autologous cultured AT-MSCs suspended in autologous inactivated serum (AT-MSC-serum) or autologous inactivated serum (serum) were injected intralesionally 2 weeks after surgical creation of centrally located SDFT lesions in both forelimbs of nine horses. Healing was assessed clinically and with ultrasound (standard B-mode and ultrasound tissue characterization) at regular intervals over 24 weeks. After euthanasia of the horses the SDFTs were examined histologically, biochemically and by means of biomechanical testing.

**Results:**

AT-MSC implantation did not substantially influence clinical and ultrasonographic parameters. Histology, biochemical and biomechanical characteristics of the repair tissue did not differ significantly between treatment modalities after 24 weeks. Compared with macroscopically normal tendon tissue, the content of the mature collagen crosslink hydroxylysylpyridinoline did not differ after AT-MSC-serum treatment (*p* = 0.074) while it was significantly lower (*p* = 0.027) in lesions treated with serum alone. Stress at failure (*p* = 0.048) and the modulus of elasticity (*p* = 0.001) were significantly lower after AT-MSC-serum treatment than in normal tendon tissue.

**Conclusions:**

The effect of a single intralesional injection of cultured AT-MSCs suspended in autologous inactivated serum was not superior to treatment of surgically created SDFT lesions with autologous inactivated serum alone in a surgical model of tendinopathy over an observation period of 22 weeks. AT-MSC treatment might have a positive influence on collagen crosslinking of remodelling scar tissue. Controlled long-term studies including naturally occurring tendinopathies are necessary to verify the effects of AT-MSCs on tendon disease.

**Electronic supplementary material:**

The online version of this article (doi:10.1186/s13287-017-0564-8) contains supplementary material, which is available to authorized users.

## Background

Tendon injuries are common in both human [1–3] and equine [4–6] athletes. In horses, the superficial digital flexor tendon (SDFT), which is located at the palmar aspect of the limb, acts to store and release elastic energy and is subject to strains close to its functional limits [7, 8]. Due to gradual accumulation of degenerative damage during intensive training leading to partial rupture, this tendon is prone to failure, especially in racehorses [5]. Because of the high incidence, prolonged recovery period and high re-injury rate, a plethora of physical, medical and surgical interventions have been applied over the years to improve quality of the repair tissue; however, to date the ideal treatment concept has not been found [9–11]. Potentially regenerative therapies, in particular cell and blood-based substrates, have gained interest over the last decade [12]. The administration of multipotent cells, in particular autologous mesenchymal stromal cells (MSCs) [13], or totipotent embryonic stem cells (ESCs) [14, 15] into tendon defects is suggested to have direct and indirect influences on tendon healing. It is thus hypothesized that the injected cells may either differentiate into cells capable of synthesizing tendon matrix—that is, have a direct regenerative effect [16–20]—or act by a paracrine effect through the release of trophic mediators, growth factors and immunomodulatory, angiogenic as well as anti-apoptotic substances [21–26].

To date it is not clear which cell source is the ideal choice to enhance tendon regeneration [27]. Comparing different adipose tissue-based cell-rich substrates, adipose-derived nucleated cells (ADNCs) are, by contrast with adipose tissue-derived mesenchymal stromal cells (AT-MSCs), a mixture of different cell types. The advantage is that ADNCs are readily available within hours after tissue harvest without the demand of cost and time for culture. Multipotency has been proven for pericytes [28], which form a subset of the ADNCs. However, AT-MSC culture leads to a higher cell dose and theoretically to a greater effect [29]. To the knowledge of the authors, however, it is not clear which cell type contributes most to tendon healing [28, 30]. In an experimental equine collagenase model study ADNCs had a limited effect on tendon healing but led to histologically improved tendon organization and an increase in cartilage oligomeric matrix protein (COMP) expression [30].

The clinically most relevant sources of MSCs in equine orthopaedics are bone marrow, adipose tissue and umbilical cord blood [27, 31]. The advantages of adipose tissue over bone marrow are that it is widely available and easily accessible, and its MSC content is higher with a higher proliferation capacity of AT-MSCs [32] and a slower senescence than that of bone marrow mesenchymal stromal cells (BM-MSCs) [27, 33, 34]. Despite the lack of a definite set of surface markers to characterize equine tenocytes, a recent study demonstrated that, compared with BM-MSCs, umbilical cord blood MSCs and AT-MSCs express collagen 1A2, collagen 3A1 and decorin at the highest levels with the highest collagen type 1A2:3A1 ratio [32]. AT-MSCs show high expressions of the tendon markers COMP and scleraxis [20, 32]. Furthermore, AT-MSCs have already been used with promising results to treat equine tendinopathies, as described in several uncontrolled case series [35–38]. In a controlled in-vivo experimental study, the implantation of AT-MSCs into collagenase-induced SDFT core lesions resulted in improved tendon fibre organization and decreased inflammatory infiltrate, as well as increased collagen type I gene expression compared with the control limbs, whereas no differences were seen in clinical parameters and with B-mode ultrasonography [39]. In another study from the same group using the collagenase-gel model of tendinopathy, intralesional treatment with AT-MSCs suspended in platelet concentrate prevented progression of SDFT lesions and resulted in better organization of collagen fibrils, less inflammation and increased vascularity [40]. However, the relevance of this model is questioned due to the strong acute inflammatory response after collagenase injection, which is unlike naturally occurring degenerative tendon lesions, and due to difficulties in standardization of lesions [41–44]. A recently introduced surgical model of centrally located SDFT (core) lesions is thought to mimic the characteristics of overuse tendinopathy more realistically [43, 45–47].

Since the 1980s, B-mode ultrasonography has been considered the gold standard for the diagnosis of tendinopathy [48, 49]. Because of its limitations, such as the lack of axial information, operator dependence, influence of ultrasound beam angle and limited resolution, however, the value of quantification of B-mode ultrasound images for the adequate assessment of repair is questioned [50, 51]. Ultrasound tissue characterization (UTC) is a new technique that was developed to analyse echo pattern stability on a computerized basis [52, 53]. Transverse ultrasound (US) images are captured at regular distances over the long axis of the tendon, and are reconstituted to a three-dimensional block of US information with the help of custom-designed software. Depending on the echo pattern stability over contiguous images, four different echo types can be discriminated with histo-morphology as a reference test [44, 52]. UTC has been shown a viable diagnostic tool to monitor experimental tendinopathies [44, 47, 54] and natural tendon disease [55, 56] in horses and is increasingly used for the assessment of (Achilles) tendon integrity in humans [57–59]. Histologic examination is still the gold standard to assess tendon healing [60] and commonly yields the most important results at the end of terminal experimental studies when findings can be correlated to those from other examination modalities (e.g. diagnostic imaging) [30, 44, 45]. Another major component of experimental tendon studies should be biomechanical testing of tendon specimens to quantify functional properties of the repair tissue which are potentially representative for the resistance to strain in a clinical setting [45, 61, 62].

To our knowledge, the effect of AT-MSCs has not been tested experimentally in horses over a 6-month period using a controlled blinded, randomized study design with non-invasive monitoring and multimodal end-stage evaluation of the repair tissue. Therefore, the aim of this study was to assess whether a single intralesional implantation of AT-MSCs suspended in autologous inactivated serum into surgically created lesions leads to a reduction of inflammatory signs and improved ultrasonographic, biochemical, biomechanical and histologic characteristics compared with the application of autologous inactivated serum alone.

## Methods

### Horses

Nine horses (eight warmbloods, one trotter) aged 3–6 years (mean 4 years) with a mean bodyweight of 545 kg (range 498–607 kg) were included in this study. All horses were housed in boxes and fed hay and cereals. Prior to the study, none of the horses showed clinical and/or ultrasonographic (B-mode, UTC) signs of forelimb SDFT disorders.

### Surgical creation of lesions and adipose tissue harvest

Core lesions were surgically created in the SDFTs of both forelimbs using the model introduced by Little and Schramme [63] and modified by Bosch et al. [45] and Schramme et al. [43]. All horses received meloxicam (0.6 mg/kg bwt (IV)) preoperatively and 2 days postoperatively. No perioperative antimicrobials were administered. A standard protocol for inhalation anaesthesia was used and the horses were positioned in lateral recumbency. After clipping and aseptic preparation, a 1.5-cm incision was made in the palmar midline, 2 cm proximal to the proximal end of the common digital flexor tendon sheath through the skin and the mesotendon into the tendon core. A 2.5-mm blunt conical obturator (Karl Storz, Tuttlingen, Germany) was inserted and guided proximally inside the tendon core under ultrasonographic guidance over a distance of 7 cm. Subsequently, a 3.5-mm burr (Abrador Burr 28200RN; Karl Storz) was inserted in the tunnel that had been created, activated and gradually pulled backwards over 20 seconds, while pressing the tendon against the tip of the burr. Care was taken not to damage the dorsal epitenon. Epitenon incisions were sutured in a simple interrupted pattern with polyglactin 910 (Vicryl® 2-0 USP; Ethicon, Norderstedt, Germany), and skin incisions were closed in a vertical mattress pattern with polyamide (Dafilon® 2-0 USP; Braun Melsungen, Melsungen, Germany). Double-layer bandages were applied and changed every 1–2 days until intralesional injection.

During the same general anaesthesia, the right paraxial gluteal region was clipped and aseptically prepared. A 4-cm skin incision was made in the cranio-caudal direction and 20 g of adipose tissue was excised from the subcutis. The skin was sutured with a vertical mattress pattern with polyamide (Dafilon® 1 USP; Braun Melsungen).

Adipose tissue was stored in MSC medium containing 1 g/l Dulbecco’s Modified Eagle’s Medium (DMEM) with 25 mM 4-(2-hydroxyethyl)-1-piperazineethanesulfonic acid (HEPES) and 1% l-glutamine (DMEM with 25 mM HEPES, with l-glutamine; PAA Laboratories, Pasching, Austria), fetal bovine serum (PAA Laboratories) and 1% penicillin/streptomycin (Penicillin-Streptomycin 100; PAA Laboratories) at 5 °C and shipped to the laboratory overnight.

### AT-MSC isolation and culture

Adipose tissue was mechanically separated using a scalpel blade and a tissue chopper. The tissue was digested with 0.05% type IV collagenase (Sigma Aldrich, Taufkirchen, Germany) and 0.025% protease (Dispase II; Sigma-Aldrich) at 37 °C for 30 min until it was neutralized by 10% fetal bovine serum (PAA Laboratories). After centrifugation (200 × *g*, 10 min), the pellet was suspended in 10 ml of Hanks’ buffer (HBSS 1× with Ca and Mg; PAA Laboratories), centrifuged again with the same settings, and the pellet containing the MSCs was suspended in MSC medium in 75-ml polypropylene flasks (T75 flask; BD Falcon, Franklin Lakes, NJ, USA) and cultured at 37 °C and 5% CO_2_. The MSCs were selected by plastic adhesion. MSCs were transfected with recombinant lentivirus particles expressing sequences of the promoting region for transcription factor hUbiC (copGFP; System Biosciences, Mountain View, CA, USA) to be able to track the MSCs via autofluorescence, which was part of another study presented elsewhere.

After 95–100% confluence of cells was reached, the medium was removed and MSCs were washed once with PBS (Dulbecco’s PBS without Ca and Mg; PAA Laboratories). Adherent cells were separated with 3 ml of Trypsin/EDTA solution (Trypsin/EDTA 1×; PAA Laboratories) for 5 min at 37 °C, which was checked by light microscopy. After neutralization with 6 ml of MSC medium, the suspension was transferred into a 15-ml polypropylene tube (Conical tubes 15 ml; BD Falcon), centrifuged (200 × *g*, 5 min) and the pellet suspended in 10 ml MSC medium. Cells were counted using a Neubauer counting chamber (Zählkammer Neubauer; LO Laboroptik, Friedrichsdorf, Germany), and 10 × 10^6^ MSCs were transferred into a new 15-ml polypropylene tube and washed with 10 ml of 1 g/l DMEM with 25 mM HEPES and 1% l-glutamine. After centrifugation, the pellet was suspended in 1 ml of autologous serum. The serum had been inactivated previously at 56 °C for 30 min. The MSC-serum suspension was transferred into a plastic tube (S-Monovette® 9 ml; Sarstedt, Nümbrecht, Germany), stored on ice, shipped overnight and kept at 4 °C until injection. AT-MSC culture and processing was performed as described previously [46].

### Immunophenotyping of AT-MSCs by flow cytometric analysis

**Fig. 1:**
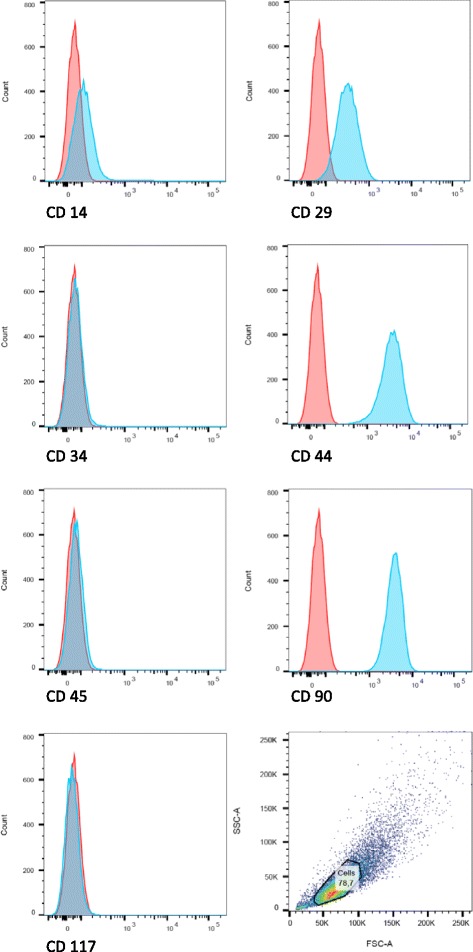
Flow cytometric analysis of cultured AT-MSCs from a representative study horse. Histograms indicate the immunophenotype of AT-MSCs for CD14, CD29, CD34, CD44, CD45, CD90 and CD117. Results are displayed for the distribution of immunostained (*green*) and unstained (*red*) AT-MSCs. All stained cells were positive for CD29, CD44 and CD90 while the signal for CD14 was weaker. No signal was detected for CD34, CD45 and CD117 (Colour figure online)

After isolation and expansion (passage 3) the AT-MSCs were incubated with monoclonal and polyclonal antibodies against CD14 (C2265-36; US Biological), CD29 (303015; Biozol), CD34 (555820; BD Pharmingen), CD44 (103005; Bio Legend), CD45 (555480; BD Pharmingen), CD90 (ab225; abcam) and CD117 (ab5616; Biozol) (Fig. 1). AT-MSCs stained only with secondary antibodies (ab150105 (abcam), DAB087583 and DAB087693 (Dianova)) were used as negative controls. Antibody binding was measured using flow cytometric analysis (BD FACSCanto™ II with BD FACSDiva™ 8.0.1 software).

### Adipogenic, osteogenic and chondrogenic differentiation of AT-MSCs

**Fig. 2:**
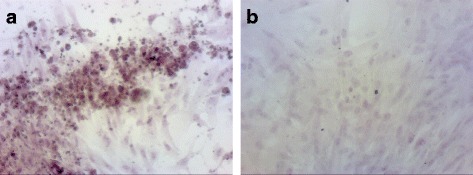
Adipogenic differentiation of equine AT-MSCs from a representative study horse. Photomicrographs of AT-MSCs (passage 2) taken 28 days after induction of adipogenic differentiation (**a**). After Oil Red staining a high number of intracellular lipid-containing vesicles was detected compared with the control without differentiation medium (**b**) (Colour figure online)

Differentiation of AT-MSCs was induced at passage 2 to adipogenic, osteogenic and chondrogenic lineages. For adipogenic differentiation, cells were seeded at a density of 2 × 10^4^ cells/cm^2^ in basal medium. After 24 hours, medium was switched to Adipogenic Induction Medium, consisting of DMEM High Glucose, supplemented with 10% FBS, 1% l-glutamine, 1% penicillin/streptomycin, 1 μM dexamethasone, 1 μM indomethacin, 500 μM 3-isobutyl-1-methylxantine (IBMX) and 10 μg/ml human recombinant insulin. Medium was changed twice a week for 14 days. Lipid production being specific for adipocytes was made visible by Oil Red staining (Fig. 2).

**Fig. 3:**
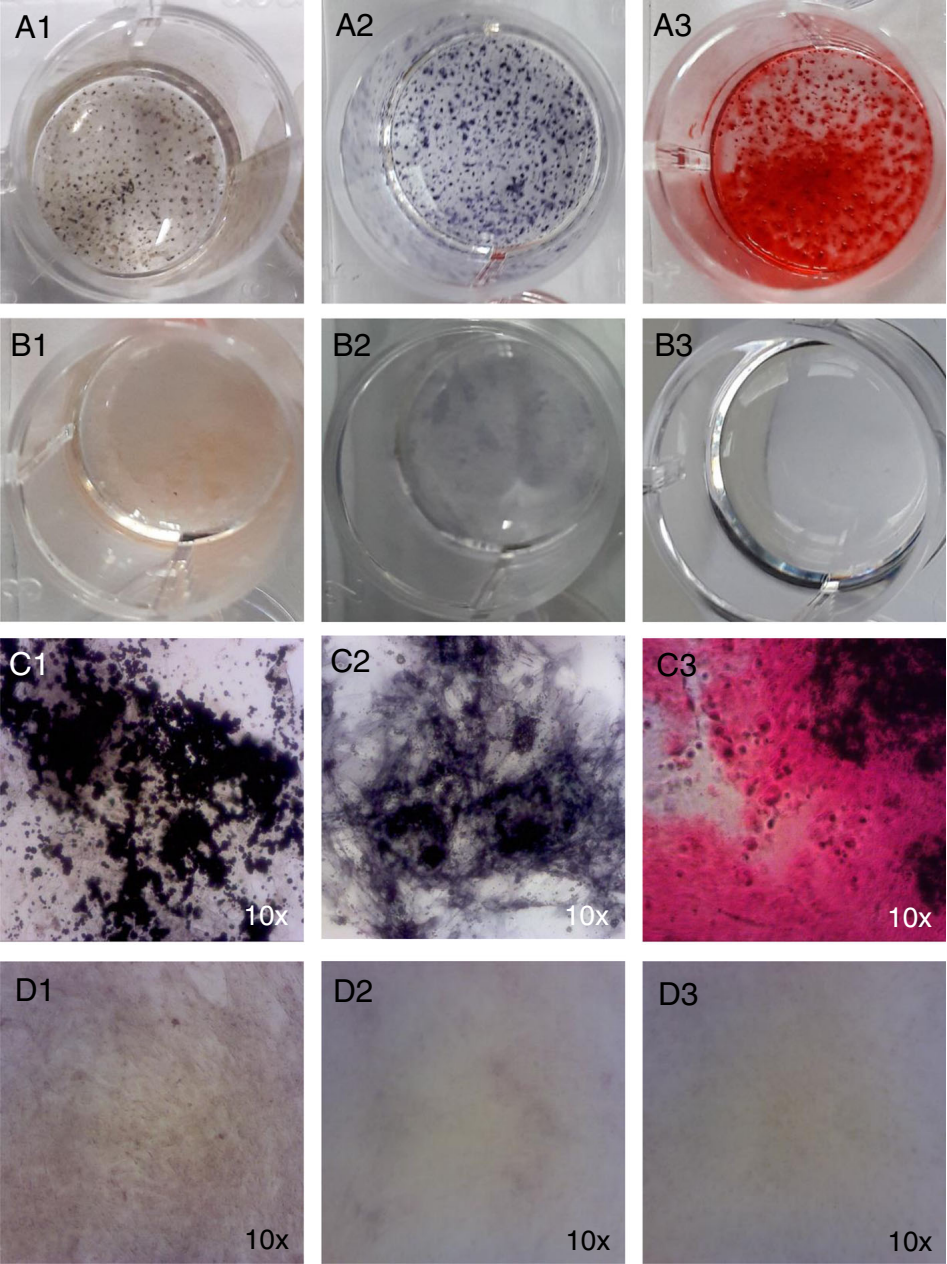
Osteogenic differentiation of equine AT-MSCs from a representative study horse. Photomicrographs of AT-MSCs (passage 2) taken on day 28 after induction of osteogenic differentiation (*A1–A3*; *C1–C3*). By contrast to controls without differentiation medium (*B1–B3*; *D1–D3*), deposition of extracellular calcium was detected by alkaline phosphatase (*A1, C1*), von Kossa staining (*A2, C2*) and Alizarin red staining (*A3, C3*) (Colour figure online)

To induce osteogenic differentiation, AT-MSCs were cultured in 24-well plates with DMEM Low Glucose with 10% FBS (MSC tested; Gibco), 1% l-glutamine (PAA Laboratories), 0.1 μM dexamethasone (Sigma Aldrich) and 1 mM β-glycerophosphate (Sigma Aldrich) for 4 weeks. Controls for osteogenic differentiation were treated under the same conditions without dexamethasone and β-glycerophosphate. The production of mineralized matrix produced by osteoblasts was made evident by alkaline phosphatase, Alizarin red and von Kossa staining (Fig. 3).

**Fig. 4:**
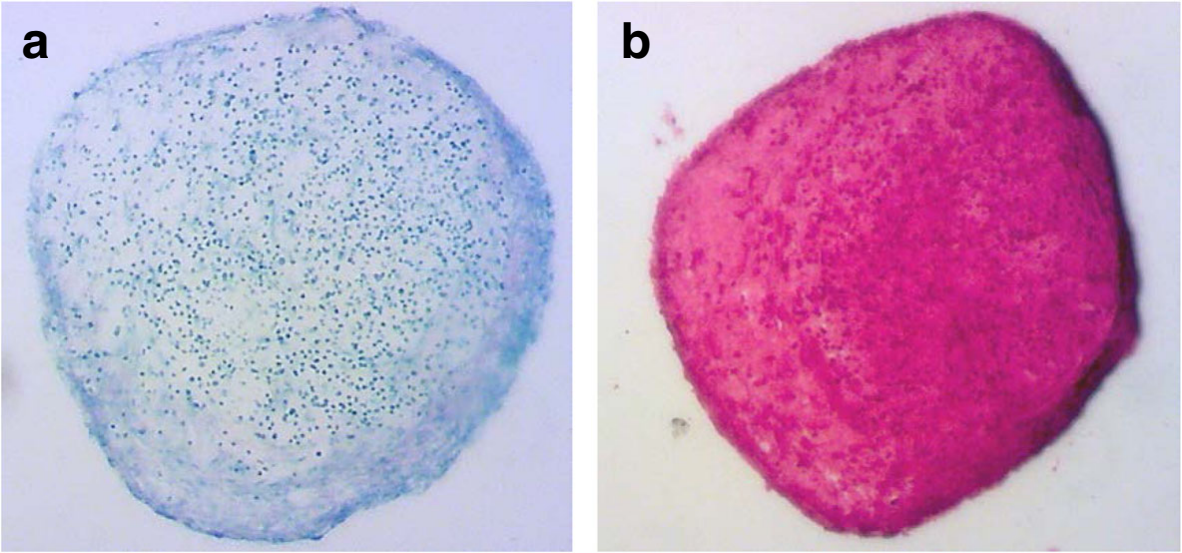
Chondrogenic differentiation of equine AT-MSCs from a representative study horse. Photomicrographs of AT-MSCs (passage 2) taken on day 21 after induction of chondrogenic differentiation. The presence of glycosaminoglycans and collagen was detected by Toluidine Blue (**a**) and Safranin O (**b**) (Colour figure online)

To demonstrate chondrogenic differentiation, the cells were trypsinized and 5 × 10^5^–1 × 10^6^ cells/ml were resuspended in DMEM High Glucose, 1% penicillin/streptomycin, 1% l-glutamine, 10% FBS, with 1× Insulin–Transferrin–Selenium (ITS) supplement, 1 mM sodium pyruvate, 100 nM dexamethasone, 40 μg/ml proline, 50 μg/ml l-ascorbic acid-2-phosphate and 10 ng/ml TGF-β1. Cells were centrifuged for 10 min at 200 × *g* in 15-ml Falcon tubes. The tubes were incubated with filter tops in a rack at 37 °C and 5% CO_2_. After 2–4 days the pellets condensed. The cells were further incubated in these tubes for 21 days. The medium was changed every 2–3 days. The production of proteoglycans being specific for cartilage was visualized with Toluidine Blue and Safranin-O staining (Fig. 4).

### Intralesional treatment of tendons with AT-MSCs

Fourteen days after creation of the lesions, horses were sedated with detomidine hydrochloride (0.015 mg/kg bwt (IV)) and butorphanol (0.025 mg/kg bwt (IV)), the hair over the palmar metacarpal region was clipped, the skin was prepared aseptically and the Nn. palmares lateralis and medialis were anaesthetized with 2.5 ml of 2% mepivacaine solution.

The core lesion of one randomly assigned SDFT of each horse was injected with 10 × 10^6^ AT-MSCs suspended in 1 ml of inactivated autologous serum, whereas the lesion in the contralateral SDFT was injected with 1 ml of inactivated autologous serum to serve as an intra-individual control. Randomization was carried out by flipping a coin and the operator was not blinded to the treatment modality.

Limbs were positioned manually to ensure equal weight bearing. For the ultrasound-guided intralesional injection, a 22-G needle was inserted from lateral at two sites (3 and 5 cm proximal to the surgical scar in the skin) and per site 0.5 ml of the inactivated serum containing AT-MSCs (AT-MSC-serum group) or inactivated serum alone (serum group) were injected intralesionally, respectively. Care was taken that the injection proceeded without resistance. A bandage was applied for 10 days and changed every second day.

### Exercise programme

All horses were subject to a standardized hand-walking exercise programme as described previously by Bosch et al. [45] (Additional file 1) on firm flat ground mainly in straight lines. Horses were turned to the left and to the right equally often. Trotting exercise was carried out on a treadmill at 3.1 m/s.

### Clinical and ultrasonographic examinations

A general clinical examination (body temperature, heart rate, respiratory rate, appetite, limb function and comfort level) was performed daily.

Preoperatively, prior to intralesional injection at 2 weeks after surgery, and 3, 4, 5, 6, 8, 10, 12, 18, 21 and 24 weeks postoperatively, limbs were assessed clinically, via B-mode ultrasonography and with UTC. SDFT swelling was determined by palpation as an increase in diameter relative to normal tendon (0 = no increase, 1 = increase by factor 1.5; 2 = increase by factor 1.5–2; 3 = increase by more than factor 2) [56, 64, 65], skin temperature over the SDFT was assessed manually (0 = no abnormality; 1 = mild abnormality; 2 = moderate abnormality; 3 = severe abnormality) and surgical skin wounds and injection sites were inspected. Lameness was evaluated at walk during the first 18 weeks post surgery, and additionally at trot from weeks 19 to 24 by an experienced equine clinician being blinded to the treated limb (five-grade score) [66].

Prior to ultrasonographic examinations, horses were sedated with romifidine (0.04–0.08 mg/kg bwt (IV)) and butorphanol (0.02 mg/kg bwt (IV)), and the hair on the palmar aspect of the metacarpus was clipped and shaved. The skin was washed with soap and degreased with alcohol, and contact gel for ultrasound examination was applied copiously. B-mode ultrasound examination was carried out with a 6–15 MHz ultrasound probe (GE ML 6-15; GE Healthcare, Wauwatosa, WI, USA) connected to a Logiq E9 (GE Healthcare) using a standoff pad and constant settings (frequency 13 MHz, gain 52, depth 25 mm, single focal zone set at 15 mm depth). The palmar metacarpus was divided into examination zones from proximal to distal in the transverse (1A, 1B, 2A, 2B, 3A, 3B, 3C) and longitudinal plane (1, 2, 3) as described earlier [67, 68]. Images were stored digitally and analysed with a DICOM workstation programme (easyIMAGE®, easyVET®; IFS Informationssysteme, Hannover, Germany). The cross-sectional area (CSA) of the SDFT was determined on all transverse images. Values for each examination zone were added to calculate the total cross-sectional area (TCSA). Echogenicity and fibre alignment were graded semi-quantitatively on longitudinal images of each zone. Echogenicity was assigned a score of 0 (normoechoic), 1 (hypoechoic), 2 (mixed echogenicity) or 3 (anechoic) and fibre alignment was graded according to the estimated percentage of parallel fibre bundles in the lesion: 0 (>75%), 1 (50–74%), 2 (25–49%) and 3 (<25%). Scores for all levels were summarized to calculate the total echo score (TES) and the total fibre alignment score (TFAS), respectively [68]. All measurements were performed by two experienced examiners blinded to treatment modalities (FR, MH). Values for TCSA and scores for TES and TFAS from both examiners were averaged.

For the UTC examination, a 10-MHz ultrasound probe (10 L5 Smartprobe; Terason Ultrasound, Teratech Corporation, Burlington, MA, USA) connected to a laptop computer (MacBook Pro® 17 inch; Apple, Cupertino, CA, USA) loaded with software for data acquisition and analysis (UTC™ Software V.1.0.1 2010; UTC Imaging, Stein, the Netherlands) was used. The probe was fixed in a motorized tracking device with built-in standoff pad (UTC-Tracker™; UTC Imaging). Settings (depth, gain, focal zone) were standardized, and all examinations were performed by the same operator (FG) with the horse bearing weight equally on both forelimbs. With the help of the tracking device, the probe moved automatically from proximal to distal at constant speed over a distance of 12 cm. Sampling of transverse images was conducted every 0.2 mm, including the surgical site as the reference point for the analysis. The compiled 600 transverse US images were reconstructed into a three-dimensional data block of US information and stored digitally until the end of the examination period. The stability of the echo pattern of corresponding pixels in contiguous transverse images was analysed (UTC2011® Analyser V1.0.1; UTC Imaging). The following echo types were discriminated: those generated by intact and fully aligned fascicles (echo type I, green), those generated by discontinuous and less aligned fascicles (echo type II, blue), those generated by a mainly fibrillary matrix with accumulation of collagen fibrils not (yet) organized into fascicles (echo type III, red) and those generated by an amorphous matrix and fluid (echo type IV, black). A 4-cm-long tendon segment from 2 to 6 cm proximal to the scar in the epitenon was selected for analysis by one examiner (FR), being blinded to the treatment modality. Within this segment, every fifth colour-coded transverse image (distance between images 1 mm) was used to place a circular cursor (∅ 5 mm) in or around the central core lesion, depending on its size. These contours were interpolated and ratios of echo types were analysed quantitatively as fractions of the determined volume. Mean values for the proportion of each echo type were calculated for all horses and for all time points. Ten scans obtained at different time points and from different horses were chosen randomly and analysed twice by the same examiner to determine the intra-observer reliability and by three examiners to determine the inter-observer reliability.

### Euthanasia and tissue harvest

Twenty-four weeks after creation of the lesion, all horses were sedated and anaesthesia was induced using a standard protocol. Thereafter, the horses were euthanized with pentobarbital (90 mg/kg bwt (IV)). Each SDFT was excised at the level of the carpus and the fetlock and the scar of the former surgical entrance to the tendon was identified. A transverse segment of 1 cm was harvested from the lateral half of the SDFT between 2 and 3 cm proximal to the entrance portal. The half-core of the lesion could easily be identified in this segment and was excised with a microtome blade and cut into three pieces that were snap frozen and stored at –80 °C for biochemical analysis. Macroscopically normal reference tissue was harvested from an equivalent 1-cm-long segment taken between 12 and 13 cm proximal to the entrance portal; that is, in the proximal metacarpal region at least 5 cm away from the proximal end of the core lesion.

The medial halves of the SDFT segments 2–7 cm proximal and 13–18 cm proximal to the epitenon scar (macroscopically normal control tissue) were obtained for biomechanical testing and stored in PBS-soaked gauze at –20 °C until analysis. The lateral half of the segment 4–6 cm proximal to the epitenon scar was collected for histological analysis, fixed in 4% paraformaldehyde for 7 days and stored in PBS at 4 °C until embedding in paraffin. Later, 5-μm longitudinal slices of the core lesion including adjacent tissue were cut starting from the centre of the tendon and stained with haematoxylin and eosin (H&E).

### Histologic examination

The centre of each tendon was independently judged by two observers blinded to horse and treatment (FG, FR). In total, five high-power fields (40× magnification) per section were examined with a light microscope (Leitz Laborlux 12; Leica, Wetzlar, Germany) using an established score [45, 60] (Additional file 2). Score values determined by each observer were calculated for each parameter before score values of both examiners were averaged.

### Biochemical analysis

#### Glycosaminoglycans and DNA analysis

After lyophilization of tendon samples, the dry weight was determined and they were digested overnight at 60 °C in 400 μl papain solution (2 mM cysteine, 1 U/ml papain, 50 mM NaH_2_PO_4_ and 2 mM EDTA, pH 6.5). A modified 1,9-dimethylmethylene blue (DMMB) dye binding assay was used to analyse the sulphated GAG concentration [45].

To a 20 μl sample, 10 μl of 3% (w/v) bovine serum albumin and 250 μl of reagent (46 μM DMMB, 40 mmol/l glycine and 42 mmol/l NaCl adjusted to pH 3.0 with HCl (hydrochloric acid)) were added, and the absorbency at 525 and 570 nm was measured after 30 min. The assay was standardized with shark chondroitin sulfate (1–100 μg/ml).

Quantification of total DNA was performed utilizing the reaction of fluorescent dye [69]. Briefly, 2 ml Hoechst 33258 (Molecular Probes, Leiden, the Netherlands) fluorescent dye solution (0.1 pg/ml in 10 mM Tris, 1 mM ethylenediaminetetraacetate (EDTA), 0.1 M NaCl pH 7.4) was added to an 80 μl sample and, immediately after mixing, fluorescence was measured using a LS-50B fluorimeter (Perkin Elmer, Norwalk, CT, USA), with excitation at 352 nm and emission at 455 nm. Salmon sperm (0–20 μg/ml) was used as a standard. All results were expressed as μg/mg dry weight tendon.

#### Collagen and crosslink analysis

Tendon samples were hydrolysed (110 °C, 18–20 h) in 600 μl of 6 M HCl for mass spectrometric determination after lyophilizing for 24 hours. Hydroxyproline (Hyp) was determined as a measure of total collagen content, and the amino acid lysine (Lys), hydroxylysine (HLys, a measure for the degree of lysine hydroxylation) and the pyridinoline crosslinks hydroxylysylpyridinoline (HP) and lysylpyridinoline (LP) as measures for the post-translational modifications of collagen. To the hydrolyzed tendon samples, 200 μl of 2.4 mM homo-arginine was added after which the samples were vacuum-dried and dissolved in 20% acetonitrile containing 1.2 mM of tridecafluoroheptanoic acid. Samples were centrifuged at 13,000 × *g* for 10 min. Supernatants were subjected to high-performance liquid chromatography (HPLC) and mass spectrometry (MS), using a 4000 Q-TRAP mass spectrometer (MDS Sciex, Foster City, CA, USA) at a source temperature of 300 °C and a spray voltage of 4.5 kV. Amino acids were separated on a Synergi MAX-RP 80A column (250 × 3 mm, 4 μm; Phenomenex Inc., Torrance, CA, USA) at a flow rate of 400 μl/min, using a gradient from MilliQ® water to acetonitrile, both containing 1.2 mM of tridecafluoroheptanoic acid and 2.5 mM ammonium acetate. Amino acids were identified by MS in multiple reaction mode using the mass transitions 429.3/82.0 (HP), 413.3/84.0 (LP), 189.2/143.7 (homo-arginine), 147.2/130.2 (Lys), 163.2/128.1 (HLys) and 131.8/67.8 (Hyp). Data were related to the recovery of internal standard. Collagen content was calculated as follows:

$$ \mathrm{Collagen}\ \left(\upmu \mathrm{g}\right) = \left[\mathrm{Hyp}\ \left(\mathrm{pmol}\right)\ /\ 300\right] \times 0.3 $$

where 300 is the number of Hyp residues in one collagen triple helix and 0.3 is deduced from the molecular weight of collagen (30,000 Da).

### Biomechanical testing

Tendon specimens were thawed at room temperature for approximately 2 hours before trimming. Two strips of 2 × 2 mm width and 50 mm length were cut from the visible lesion and a single strip was harvested from a tendon segment with macroscopically normal tissue (further proximal to the lesion site) from each tendon using a custom-designed cutting device [45, 70]. Specimens were marked and stored in PBS-soaked gauze at 4 °C and tested within 2 hours. Before testing, the exact CSA was measured at four locations along the length of the specimen using a laser device (Laser Micro Diameter LDM-110; Takikawa Engineering, Japan) and the average CSA was calculated. Strips were fixed in the loading device of a universal materials testing machine (Zwick 1445; Zwick, Germany) in a PBS bath and preconditioned with 3% strain at 1 Hertz for seven cycles. After that, specimens were loaded to failure with 0.5 mm/s. For each specimen, force (F) and displacement were recorded. The stress at failure (σ_max_) was calculated as follows:

$$ {\upsigma}_{\max }={\mathrm{F}}_{\max }/\ \mathrm{C}\mathrm{S}\mathrm{A}\ \left(\mathrm{N}/{\mathrm{mm}}^2 = \mathrm{MPa}\right) $$

and the modulus of elasticity was derived from the linear part of the stress–strain curve [45, 61]. Results of the two specimens taken from macroscopically normal tissue (left and right SDFTs) were pooled in one group.

### Statistical analysis

Clinical, B-mode ultrasound, UTC, histology and biochemistry data were analysed using SAS® 9.3 (SAS Institute, Cary, NC, USA). The assumption of a normal distribution of quantitative parameters was examined using the Shapiro–Wilk test and visual assessment of distributions. In normally distributed samples, parametric methods were used: *t* test for paired observations and analysis of variance for calculation of variance components (intra-class correlation coefficient (ICC)). Otherwise, non-parametric tests (Kruskal–Wallis test and Wilcoxon two-sample test) were applied.

Data from biochemical testing, which were partly not normally distributed, were tested using the Wilcoxon signed-rank test (R version 3.0.2; The R Foundation for Statistical Computing, Vienna, Austria). The Kruskal–Wallis test was used to compare CSAs of specimens, stress at failure and modulus of elasticity among all three tested groups. Individual comparisons of the three groups (AT-MSC-serum, serum, macroscopically normal tissue) were performed using a Wilcoxon signed-rank test with Bonferroni correction for multiple testing.

A significance level of α = 0.05 was applied. Intra-observer and inter-observer repeatability of UTC measurements and histology scores were calculated using the ICC. A repeatability value > 0.75 was considered excellent, 0.75–0.4 as fair to good and <0.4 as poor [71]. Proc NESTED and Proc VARCOM were used for calculation of the coefficient of variance.

## Results

### Lesion creation, adipose tissue harvest and intralesional treatment

Surgical creation of core lesions was successful in all limbs; the epitenon was not damaged inadvertently in any case. Harvest of adipose tissue, isolation and culture of AT-MSCs was carried out without complications. Immunophenotyping and trilineage differentiation of cultured cells showed typical characteristics of MSCs (Figs. 1, 2, 3 and 4) [72].

Intralesional injections were uneventful in all horses.

#### Clinical examination

No lameness was evident at walk until week 17 and at walk and trot from week 18 to 24. All horses developed an increase in skin temperature over the SDFT during the course of the experiment, without differences between the groups. Mild to moderate swelling of the palmar metacarpal region was palpable in both groups after induction of lesions. This swelling decreased markedly until 3 weeks and increased markedly again until week 8 after injection in all limbs. Scores for palpable swelling remained consistently high in the serum group, while they decreased continuously in the AT-MSC-serum group. This difference was weakly significant (*p* = 0.0497) at 21 weeks after surgery. At the end of the observation period, palpable SDFT swelling was mild in all horses. Two horses developed cellulitis in the region of the surgical entrance, which healed without pharmaceutical intervention.

### B-mode ultrasonography

**Fig. 5:**
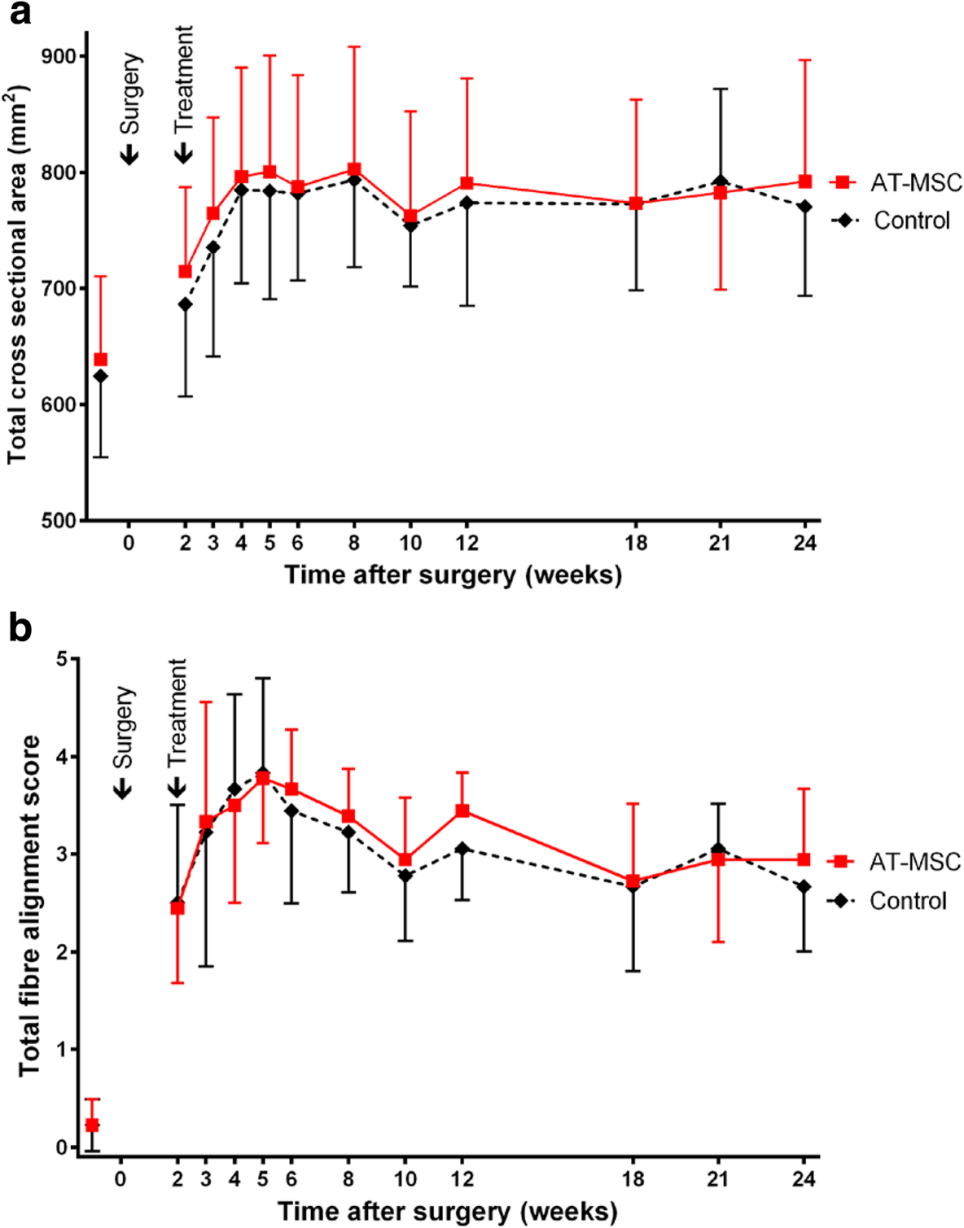
B-mode ultrasonographic parameters. Development of ultrasonographic scores (mean ± SD) of SDFTs treated with AT-MSCs suspended in autologous inactivated serum (*solid lines*) and a﻿utologous inactivated serum alone (*dotted lines*) over 24 weeks after surgical induction of tendon injuries. **a** TCSA. **b** TFAS. *AT-MSC* adipose-derived mesenchymal stromal cell

All horses developed bilateral tendon core lesions with an ultrasonographic morphology similar to naturally occurring tendon disease. There were no significant differences in TCSA, TFAS (Fig. 5) and TES between the groups at any point in time. Mean TCSA increased markedly in both groups until 4 weeks and reached its maximum at 8 weeks after lesion induction (AT-MSC-serum, 803 mm^2^, serum, 793 mm^2^). TFAS increased similarly and peaked at week 5 after lesion induction (Fig. 5). TCSA and TFAS decreased again until week 10.

### Ultrasound tissue characterization

Intra-class correlation for UTC measurements was excellent for intra-observer reliability (0.99) and inter-observer reliability (0.80–0.97 depending on echo type).

**Fig. 6:**
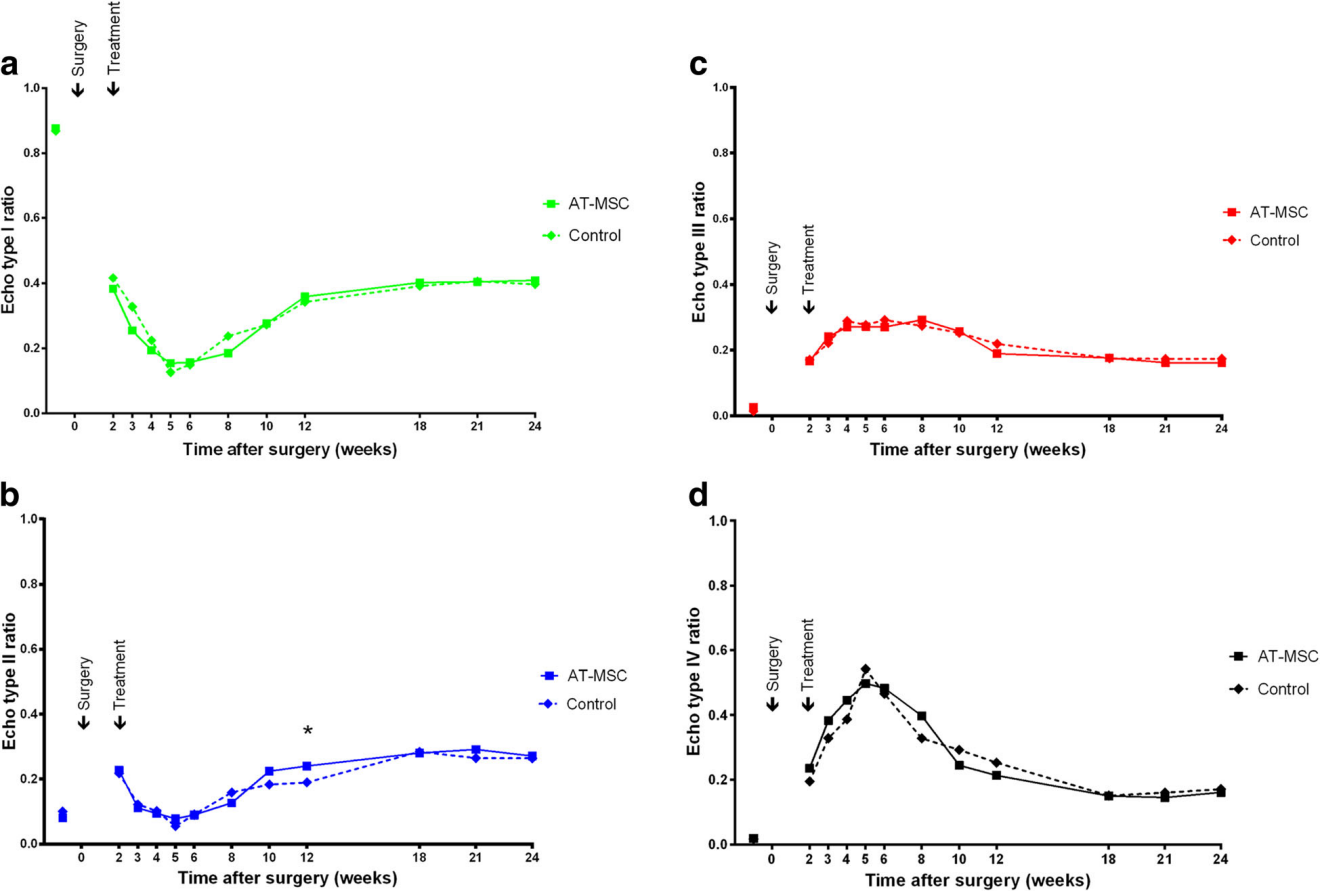
Ultrasound tissue characterization. Development of echo type ratios for SDFTs treated with AT-MSCs suspended in autologous inactivated serum (*solid lines*) and autologous inactivated serum alone (*dotted lines*) over 24 weeks after surgical induction of tendon injuries. **a** Echo type I. **b** Echo type II. **c** Echo type III. **d** Echo type IV. *Significant difference between groups. *AT-MSC* adipose-derived mesenchymal stromal cell

The development of echo type ratios within SDFT lesions over time is shown in Fig. 6. There was a strong decrease of structure-related echo types I and II until week 5 after lesion induction (Fig. 6a, b) and a concomitant increase of non-structure-related echo types III and IV in both groups (Fig. 6c, d), indicative for tissue damage, loss of structural organization and inflammatory response. Echo type III ratios indicating fibrillogenesis peaked between weeks 6 and 8 postoperatively in both groups. Echo type II ratios increased from weeks 6 to 18, which is indicative for a fibrillary matrix being organized into fascicles that are not yet aligned properly. The significantly higher ratio of echo type II in the AT-MSC-serum group in week 12 post surgery (*p* = 0.0326) may be indicative for increased remodelling (Fig. 6b). Simultaneously there was an increase in echo type I ratios without any differences between the groups. From week 12 post surgery onwards, all echo type ratios did not change markedly until the end of the study.

### Gross pathologic examination

**Fig. 7:**
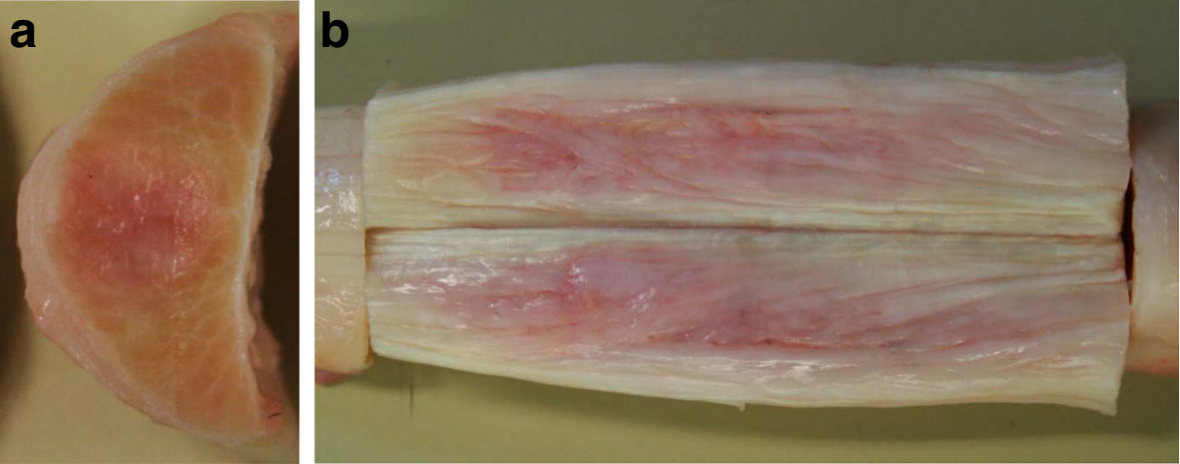
Gross pathologic examination of SDFTs. **a** Transverse and **b** longitudinal section of a serum-treated tendon segment 24 weeks after surgical induction of tendinopathy, 2 and 2–5 cm proximal to the surgical entrance into the tendon, respectively. The centrally located scar tissue is pale to intensively pink, partly reddish and well demarcated from surrounding ivory-coloured tendon tissue. The scar is nearly circular in shape on the transverse and appears as an oblong area on the longitudinal section (Colour figure online)

At post-mortem gross examination, injection sites were still visible as two unilateral swellings of the lateral aspect of the tendon. Scar tissue was visible within the centre of the tendons as the core lesion (Fig. 7). Macroscopically, there were no differences visible between the groups.

### DNA, GAG, collagen and crosslink content

The dry weight of samples from AT-MSC-serum-treated and from serum-treated tendon lesions as well as samples from macroscopically normal tissue from the same tendons did not differ significantly (*p* > 0.05). The contents of GAG, DNA, Hyp, total collagen, HP, LP and HLys did not differ between lesions from the AT-MSC-serum group and the serum group. Tendon lesions treated with AT-MSC-serum and those treated with serum alone contained both more GAG (AT-MSC-serum, *p* = 0.0078; serum, *p* = 0.0039) and DNA (AT-MSC-serum, *p* = 0.0039; serum, *p* = 0.0039) and less Hyp (AT-MSC-serum, *p* = 0.0195; serum, *p* = 0.0078) and total collagen (AT-MSC-serum, *p* = 0.0195; serum, *p* = 0.0078) than macroscopically normal tissue from the same tendons (Table 1). LP and HLys content was the same in tendon lesions treated with AT-MSC-serum and serum alone and in macroscopically normal tissue (*p* > 0.05). Tendons treated with AT-MSC-serum had the same HP content as tendons treated with serum alone (*p* = 0.25) and macroscopically normal tendon tissue (*p* = 0.0742). By contrast, normal tendon tissue contained more HP than tendon treated with autologous inactivated serum alone (*p* = 0.0273).

**Table 1:** Biochemical parameters of SDFTs treated with AT-MSCs suspended in autologous inactivated serum (AT-MSC-serum) and autologous inactivated serum alone (serum) and macroscopically normal tendon tissue (normal) from the same tendons 22 weeks after treatment

	Group		
Parameter	AT-MSC-serum (*n* = 9)	Serum (*n* = 9)	Normal (*n* = 18)
DNA (μg/mg dwt)	3.91 ± 0.96^a^	4.24 ± 1.31^a^	1.99 ± 0.30^b^
GAG (μg/mg dwt)	21.38 ± 11.44^a^	26.80 ± 10.26^a^	6.47 ± 1.16^b^
Hyp (mg/mg dwt)	0.067 ± 0.001^a^	0.062 ± 0.012^a^	0.083 ± 0.009^b^
Total collagen (mg/mg dwt)	0.510 ± 0.076^a^	0.469 ± 0.093^a^	0.635 ± 0.071^b^
HP (mol/mol col)	0.196 ± 0.025^ab^	0.184 ± 0.026^b^	0.238 ± 0.040^a^
LP (mol/mol col)	0.016 ± 0.005^a^	0.017 ± 0.007^a^	0.013 ± 0.005^a^
HLys (mol/mol col)	6.90 ± 1.76^a^	7.39 ± 2.37^a^	7.38 ± 1.96^a^

Values presented as mean ± standard deviation

*col* collagen, *dwt* dry weight, *GAG* glycosaminoglycans, *HLys* Hydroxylysine, *HP* hydroxylysylpyridinoline, *Hyp* Hydroxyproline, *LP* lysylpyridinoline, *AT-MSC* adipose-derived mesenchymal stromal cell, *SDFT* superficial digital flexor tendon

^a,b^Different superscript letters indicate significant differences (*p* < 0.05) between treatment groups

### Histology

**Fig. 8:**
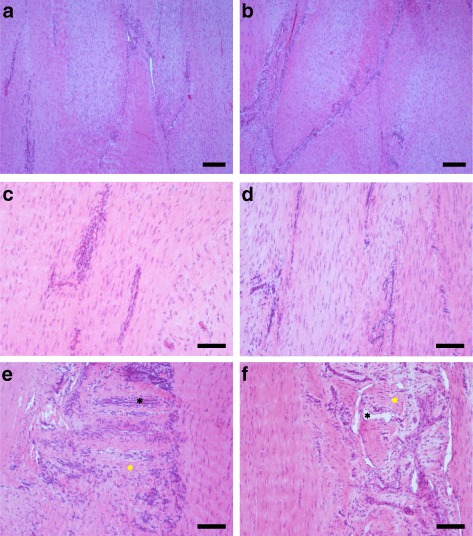
Histology of surgically induced SDFT lesions 22 weeks after treatment. **a**–**f** Longitudinal specimens of tendon lesions treated with AT-MSCs suspended in autologous inactivated serum (**a**, **c**, **e**) and autologous inactivated serum alone (control; **b**, **d**, **f**) stained with H&E (**a**, **b**, *scale bar* = 200 μm; **c**, **d**, **e**, **f**, *scale bar* = 100 μm). Fibril arrangement was mostly unidirectional (**a**–**d**), with some cases showing large regions without any regular fibre arrangement (**e**, **f**; ye*llow asterisk*) in both groups. Specimens showed variations in cell density (**a**–**d**) and regions with high cellularity and vascularization after both treatment modalities (**e**, **f**; *black asterisks*) (Colour figure online)

**Fig. 9:**
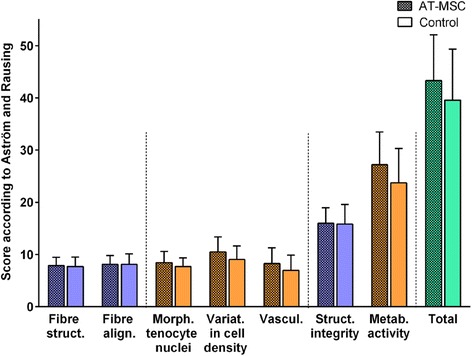
Histomorphological scores (﻿As﻿tröm﻿ and Rausing [60], modified by Bosch et al. [45]﻿). Mean histomorphological scores (± SD) for surgically induced SDFT lesions treated with AT-MSCs suspended in autologous inactivated serum (AT-MSC) and with autologous inactivated serum alone (control) 22 weeks after treatment. Total scores (*green*) include values from sub-scores for fibre structure (*struct*.), fibre alignment (*align*.), morphology (*morph*.) of tenocyte nuclei, variations (*variat*.) in cell density in cell density and vascularization (*vascul*.). Scores for structural (*struct*.) integrity and metabolic (*metab*.) activity summarize values from sub-scores displayed in *blue* and *red*, respectively. Scores and sub-scores did not differ between treatment modalities (*p* < 0.05). *AT-MSC* adipose-derived mesenchymal stromal cell (Colour figure online)

Intra-class correlation for histology scores was excellent for inter-observer reliability (0.77–0.97 depending on score/sub-score). The lesions could be identified clearly on all H&E-stained slices. Histology was indicative for an incomplete restoration of structural integrity and a high metabolic activity (Fig. 8). There were no significant (*p* < 0.05) differences between the AT-MSC-serum and serum groups alone with respect to total scores, scores for fibre arrangement, scores for metabolic activity and sub-scores for fibre structure, fibre alignment, morphology of tenocyte nuclei, variations in cell density or vascularization. Scores for structural integrity and metabolic activity remained high after the 24-week observation period (Fig. 9).

### Biomechanical testing

**Fig. 10:**
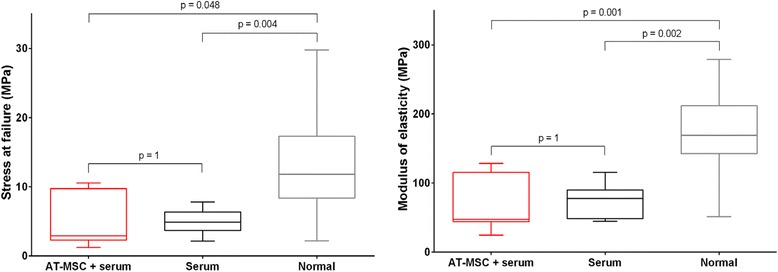
Biomechanical parameters. Stress at failure and modulus of elasticity (measure of tensile stiffness of materials) of surgically induced SDFT lesion tissue treated with AT-MSCs suspended in autologous inactivated serum (*AT-MSC + serum*) and autologous inactivated serum alone (*serum*) 24 weeks after creation of lesions and 22 weeks after treatment. Macroscopically normal tendon tissue (*normal*) was harvested from a proximal segment of the same tendons. *AT-MSC* adipose-derived mesenchymal stromal cell

Cross-sectional areas of tendon strips were not significantly different between AT-MSC-serum-treated lesion tissue, serum-treated lesion tissue and macroscopically normal tendon tissue (*p* = 0.11). Stress at failure and modulus of elasticity did not differ between AT-MSC-serum-treated tendons and those treated with serum (*p* = 1) (Fig. 10). Compared with macroscopically normal tendon tissue, stress at failure and modulus of elasticity were significantly lower in AT-MSC-serum-treated lesion tissue (*p* = 0.048 and *p* = 0.001, respectively) as well as in serum-treated lesion tissue (*p* = 0.004 and *p* = 0.002, respectively).

## Discussion

Standardized central lesions were successfully induced in the SDFT of horses and longitudinally followed using standard and more sophisticated monitoring techniques for 24 weeks before in-depth histological, biochemical and biomechanical tissue analysis.

The current study shows that a single treatment with 10 × 10^6^ AT-MSCs suspended in inactivated autologous serum does not have a lasting effect on signs of inflammation and does not substantially improve ultrasonographic histologic, biochemical or biomechanical characteristics of surgically created SDFT core lesions over a 24-week period compared with inactivated autologous serum alone. However, the fact that the hydroxylysylpyridinoline (HP) content in the AT-MSC-serum treatment group is closer to the normal situation may potentially indicate improved collagen crosslinking.

Hydroxylysine (HLys) is produced during a post-translational enzymatic modification process in tendon and is needed for the formation of the mature crosslinks HP and LP [73, 74]. Newly formed collagen within a lesion is less crosslinked than in mature tissue, which was most likely caused by enzymatic cleavage after the surgical trauma and the subsequent formation of more stable crosslinks during the remodelling phase resulting in repair tissue with higher tensile strength and stiffness [75].

The HP crosslink concentration per molecule of collagen in our study was the same in normal tissue as in AT-MSC-treated lesion tissue while lesion tissue treated with inactivated serum alone contained significantly less HP than macroscopically normal tissue. Despite the lack of significance between the AT-MSC-serum and serum groups this might be interpreted as a sign of superior crosslinking; that is, a better or more advanced repair after AT-MSC treatment.

The contents of lesion tissue in HLys and in LP as expressed per molecule of collagen were the same in tendon lesions treated with AT-MSC-serum and serum alone and in macroscopically normal tissue, showing that these post-translational modifications were by contrast not affected by creation of the lesions and the treatment modalities.

An increased GAG content of healing tendon lesions reflects a high tenocyte metabolism and an increased production of extracellular matrix. There is controversy about increased GAG content in tendon repair: on the one hand, degenerated tendon regions including fibrous scar tissue have been shown to contain higher concentrations of sulphated GAGs than normal tendons in humans and horses [76–78]. On the other hand, an increased GAG content as expressed per DNA has been interpreted as a sign of improved tendon healing in a recent equine study after intralesional platelet-rich plasma (PRP) treatment [45]. Interestingly, GAG contents of saline-treated control tendons in the latter study and in another study testing a plasma product [79] were similar to the GAG content in both groups of the current study using the same surgical model of tendinopathy, respectively. This also corresponds well to findings in naturally injured SDFTs that were saline treated and contained significantly more GAG than corresponding BM-MSC-treated as well as untreated control tendons, which were relatively uninjured [13]. In the current study, GAG contents in tendon lesions were higher than in normal SDFT tissue from more proximal regions of the injured tendons. These GAG contents in turn were very similar to those from mid-metacarpal SDFT tissue from mature horses in another study [80], suggesting that the proximal metacarpal region is suitable as a control to compare biochemical parameters of mid-metacarpal SDFTs and that SDFTs did not reach normal GAG content after treatment at the end of the observation period in the current study [80, 81], which implies that the end stage of healing had not been reached.

In the current study, lesions treated with AT-MSCs had the same content in total collagen and Hyp as tendons treated with autologous inactivated serum alone. Although replacement of tenocytes with new ones resulting from tenogenic differentiation after engraftment of MSCs with consecutive production of extracellular matrix (e.g. collagen, GAG) is a potential mechanism of the therapy used [28], the findings do not support that this mechanism of action played a major role in the current study.

Collagen and Hyp contents, however, were significantly lower than in macroscopically normal tendon tissue which contained as much collagen as unaltered tissue in a previous study [82]. The finding that lesion tissue contains less collagen than normal tendon is in accordance with a previous study using the same model [47].

Collagen types could have been differentiated between the more elastic type I and type III in the current study. However, MSCs as well as autologous serum have the potential to influence the expression of these collagen types during tendon repair [32, 62, 65]. The time frame in which expression of the collagen type I to III ratio reaches its optimum during the remodelling phase has not been clearly defined [41], so it remains unclear whether differentiation of collagen types would have helped to show which treatment modality was more beneficial in the current study.

Collagen content correlates positively with the modulus of elasticity representing stiffness and with the stress at failure of the tendon strips in the current study, because both parameters were significantly lower in lesion tissue than in macroscopically normal tendon tissue. This is a typical characteristic of scar tissue with low maturity [61] and also proves that tendon healing was still ongoing at the terminal stage of the current experiment. It has to be concluded that AT-MSCs did not significantly influence the biomechanical properties in surgically created SDFT lesions 22 weeks after treatment, as did PRP in a similar model [45]. Stress at failure, one of the most important indicators for tensile strength of tendons [61], was significantly lower after AT-MSC-serum treatment and after treatment with serum alone than in macroscopically normal tendon tissue harvested from a proximal segment from the same tendons. Based on the knowledge that intact tendon tissue from the proximal metacarpal region normally has similar biomechanical properties as tissue from the mid-metacarpal region [83], this finding suggests that scar tissue has not regained its original strength 22 weeks after AT-MSC treatment. Findings from the present study show that it is beneficial to include macroscopically normal tissue as a second control group during biomechanical testing as an intra-individual reference for normal tendon tissue which was not reported in similar studies previously [45, 79].

Biomechanical properties of the repair tissue are closely related to functionality [45, 61, 84], which becomes clinically manifest by the recurrence rate of natural tendon disease in horses and to a lesser extent by persisting lameness [41]. Horses in the present study did not show signs of lameness until the end of the study period, which is in accordance with the observation that, in contrast to human patients with Achilles tendinopathy, chronic tendon pain does not play a major role in horses [23]. However, the sensitivity of clinical examination alone is limited to monitoring lameness, especially in cases of bilateral tendon injury [3]. This could have been improved by using computerized gait analysis, but this was not available during the experiment. The study period and the exercise regimen could have been extended to determine the recurrence rate of tendinopathy, one of the most reliable parameters of long-term functionality. However, this was considered ethically unacceptable in an experimental study.

Swelling of SDFTs, another clinical parameter of inflammation, was less (weak significance) in AT-MSC-treated tendons than in control tendons only at a single time point (i.e. at 21 weeks after lesion induction). Because the ultrasonographically determined tendon cross-sectional area (TCSA) did not differ between groups at any time, the findings during palpation might be attributed to a decrease in subcutaneous swelling rather than in tendon diameter. Swelling was determined by palpation, a diagnostic modality which is subject to some inaccuracy, so it remains questionable whether AT-MSCs really influenced subcutaneous swelling during the remodelling phase (e.g. by reflux of the cell suspension during treatment).

In the current study, no significant reduction of clinical signs of inflammation and no reduction of fluid and/or cell accumulation during ultrasonography were recorded after injection of AT-MSC-serum during the acute inflammatory phase when compared with the effect of serum alone, which corroborates earlier in-vivo findings [39] - although an increased echogenicity of collagenase-induced SDFT lesions was seen after AT-MSC application in a later study from the same group [40]. Another experimental in-vivo study using the same surgical model as in the current study showed a significant reduction of fluid and cell accumulation by means of UTC early after intralesional treatment with PRP, which was interpreted as a reduction of inflammation [45]. The authors of the current study would have expected similar effects after AT-MSC injection, because these cells are known to exert anti-inflammatory effects [85–87]: human AT-MSCs led to a reduced inflammatory response in synovial cells from patients with osteoarthritis by inhibition of pro-inflammatory cytokines such as IL-1β and IL-6 [85]. Recently it has been shown in a canine model of tendon transection that collagen sheets seeded with AT-MSCs promote an anti-inflammatory M2-type macrophage phenotype [87]. Immunomodulatory actions of MSCs are thought to be dose and time dependent [29], so that a higher MSC dose, implantation at an earlier or later time point after lesion creation or repeated injections might have led to different results. To the authors’ knowledge, no experimental data exist concerning the ideal dose and timing of MSC implantation [28, 88]. On the one hand, it is hypothesized that there is a direct positive dose–effect relation [89]; on the other, experimental data show cytotoxic effects of high MSC doses [90]. In a rabbit experimental study, BM-MSC–collagen composites were used to repair patellar tendon defects and MSC concentrations of 4 × 10^6^ and 8 × 10^6^ cells/ml did not lead to additional biomechanical and histological improvement compared with 1 × 10^6^ cells/ml [91]. In the current study, the cell dose of 10 × 10^6^ AT-MSCs was based on recommendations from previous clinical and experimental trials [23, 39, 92]. The time of injection might have been too late to induce early anti-inflammatory effects, which were achieved with PRP injections as early as 7 days after lesion creation in the same model of tendinopathy [45]. Although the ideal point of time for MSC treatment is not known [28], it is hypothesized that tendons should be treated after the initial inflammatory phase and before fibrous tissue has formed [88]. Advised times range from 7 to 30 days between lesion creation and treatment with adipose tissue-derived cells has been described in experimental studies [30, 39, 40]. Using high numbers of pure MSCs for treatment, cell culture per se implies a delay of cell injection. In a large clinical study, horses treated <5 weeks after injury had a lower re-injury rate than horses treated later, but this difference was not significant [93].

In the current study the authors decided to postpone the MSC injection for another week, because lesions seemed to be less developed at ultrasonography 7 days after induction than in previous studies using the same model [43, 45, 47], which might be attributed to the use of a different burr during creation of the lesions, to confinement of the horses to box rest instead of exercising them after lesion induction, or to the model itself. The surgical model ideally creates a compartment similar to that typically seen in degenerative strain-induced tendon disease [43, 47]. Creating these lesions requires general anaesthesia, which is a disadvantage, but this model is better standardized and considered less painful for horses than induction of core lesions with collagenase, a model which rather reflects the inflammatory component of tendinopathy. The response to these interventions in terms of the expansion of the lesion seems to be less pronounced in the surgical model [42, 43]. The inflammatory response in the current study was most prominent relatively late - that is, at 5 weeks after surgery and 3 weeks after cell injection, as shown by UTC (peak in echo type IV) - so that at this time potential anti-inflammatory effects ascribed to AT-MSCs [85–87] might already have been weaker than the endogenous inflammatory response. In other words, the AT-MSCs could possibly not exert their effect adequately in an environment of only mild to moderate inflammation at the time of injection. Interestingly, in another in-vivo study using a collagenase model, AT-MSCs which were implanted 30 days after lesion creation (i.e. approximately 2 weeks later than in the current study) led to decreased inflammatory cell infiltration as determined by histology of biopsies at 30 and 120 days after treatment [39]. This finding suggests that later AT-MSC implantation at the end of a pronounced inflammatory phase might be more efficient, but the significant differences between both experimental models of tendinopathy may also play a role here.

Depending on the mode of action of MSCs implanted into tendon lesions (i.e. paracrine effects versus de-novo synthesis of tendon tissue), a decline of MSC population over time during the inflammatory and proliferative phase may be an explanation for the lack of substantial differences between the AT-MSC and control groups. Death of the MSCs is a potential reason: in an equine surgical model of tendinopathy, less than 5% of BM-MSCs survived more than 10 days and only 0.02% survived over 90 days after implantation [14]. Allogeneic embryonic stem like cells (ESCs), by contrast, were detectable at a constant level over 90 days in the same study, which shows that development of stem cells from different sources may vary after implantation. High numbers of AT-MSCs could be detected with different modalities as long as 9 weeks after implantation into surgically created SDFT lesions [94]. Labelled AT-MSCs injected into experimentally induced equine tendon lesions were partly found to remain viable and integrated in the lesion tissue after 24 weeks, although numbers of cells decreased over time, and MSCs could even be detected in SDFT lesions of the contralateral limb [95]. Together with the observation that labelled cells were retrieved in the peritendinous tissue near the injection site [94, 95], this implies a potential effect of the MSC treatment on the contralateral defect and a loss of MSCs effectively available in the treated lesion. Both effects may have limited the effect of the treatment.

It would have been valuable to test in the current trial whether the therapy exerted an anti-apoptotic effect on tenocytes, an effect which has been attributed to AT-MSCs [87]. However, to address this it would have been necessary to take tendon biopsies to perform immunostaining with markers for apoptotic cells during the inflammatory phase, given the duration of the study. This was discarded because of the potential impact of the procedure on tendon healing. Further, the attraction of precursor cells as another potential mechanism of AT-MSC therapy is more relevant during early phases of tendon healing. Even after harvesting of tendon biopsies, it would have been a challenge to estimate this effect merely via determination of the cellularity, because there is no precursor cell-specific marker.

For injection, cells must be suspended in a medium, which may consist of phosphate-buffered saline (PBS) [30] or autologous blood serum, the latter being considered a more adequate suspension medium by some authors [14, 96]. However, it was shown that autologous serum, cultivated in glass tubes at 37 °C for 24 hours, contains significant amounts of IL-1ra and IL-10 [97], which implies that serum alone might influence tendon healing [65]. Instead of fresh serum [39], thermally inactivated autologous serum was used in the present study and also injected into control group lesions. Alternatively, control lesions could have been left without puncture and treatment, but puncture alone has been shown to support drainage of early fluid accumulation and could theoretically guide peritendineal precursor cells into the lesion and thereby have a therapeutic effect [98, 99]. For the same reason, repeat biopsies were not used in the current study compared with previous studies [40]. Only two more control groups—one without any puncture, and one with puncture alone but without injection—could have ruled out these effects. However, a much larger study population would have been needed.

Twelve weeks after surgery, the ratio of type II echoes representing discontinuous fascicles not yet aligned into lines of stress was significantly higher in AT-MSC-treated lesions than in control lesions, which might be indicative for a pro-aligning influence of implanted AT-MSCs on the early organization of tendon matrix into tendon bundles [44, 54], potentially mediated by direct or paracrine coordinating effects of AT-MSCs on fibroblasts. However, differences did not last until the end of the observation period, and no effects on the percentage of type I echoes (i.e. intact tendon tissue) were evident in the AT-MSC group, so it remains unclear whether the finding is biological reality or due to chance. Clinical relevance seems limited.

Ratios for type I echoes, representative for fully aligned tendon bundles, did not reach those for intact tendon tissue inside the core lesion in terminal UTC scans. This indicates that there was still no complete restoration of perfectly aligned tendon bundles after 24 weeks, as expected after this timeframe [82]. The finding that ratios of all echo types only changed mildly in the current study after week 12 is different from that of Bosch et al. [45], who used a similar model of tendinopathy and found further improvement; that is, a more pronounced decrease of the echo type IV ratio and an increase of the echo type I ratio between weeks 12 and 24 after PRP treatment of surgically induced tendon lesions.

Results of histology, biochemical analyses and biomechanical testing, which were only performed post mortem 22 weeks after treatment, correlate well with the UTC findings observed previously [44, 45]. None of these diagnostic modalities showed differences between AT-MSC-serum-treated tendons and serum-treated controls. Compared with macroscopically normal SDFT tissue from the same horses there were significantly higher DNA and GAG contents, and lower Hyp and total collagen and HP contents. At the same time tenocyte nuclei were rounded, showing the more tenoblastic cell type, and cell density showed high local variations. Also, histologic scores for fibre structure and fibre alignment were increased in both groups. All of these findings are indicative for relatively high metabolic activity and ongoing tissue repair and remodelling at the end of the observation period (i.e. in the remodelling or maturation phase) [41]. It can be argued that to evaluate the end stage of tendon repair an observation period of 1 year would have been even more appropriate [82]. However, it had been shown previously that the phases of early remodelling or maturation when newly formed collagen type I fibres are organized into bundles and orientate themselves into lines of stress take place between approximately days 45 and 120 after lesion induction [100], and it is hardly conceivable that differences would have been noted at 1 year but not after 24 weeks.

In AT-MSC-treated lesions, no difference in vascularization was found histologically using a subjective five-point scale in this study. However, in a more detailed analysis reported elsewhere [46] in which all clearly identifiable vessels on the entire tissue section were counted in the same specimen, there was a significantly higher number of vessels in AT-MSC-treated versus control tendons. This finding, together with the increase in Doppler signal detected at 2 weeks post treatment [46], shows that the methodology used in the current study was obviously not sensitive enough to detect these relatively minor differences. In other equine experimental studies, an increase in Doppler signal could be detected at 6 weeks after implantation of AT-MSCs suspended in platelet concentrate into artificial SDFT lesions [40], whereas intralesional PRP injection led to an almost continuous increase in Doppler signal in another investigation [101]. Stimulation of angiogenesis by AT-MSCs was also postulated by several authors [22, 86] while others [90] showed endothelial cell apoptosis and capillary degeneration after application of rat BM-MSCs in vitro. Neovascularization is of utmost importance to assure transportation of cells and growth factors towards and away from the lesion site during the inflammatory and proliferative phases of extrinsic tendon healing [41]. However, there is controversy about the effects of a prolonged increase in vascularity, which is discussed elsewhere [46, 60, 102, 103].

## Conclusions

The effect of a single intralesional injection of cultured AT-MSCs suspended in autologous inactivated serum was not superior to the treatment of surgically created SDFT lesions with autologous inactivated serum alone in a surgical model of tendinopathy over an observation period of 22 weeks. AT-MSC treatment might have a positive influence on collagen crosslinking and therefore possibly on tensile stress resistance of remodelling scar tissue. Ultrasound tissue characterization (UTC) was a viable tool to monitor tendon healing non-invasively and the findings correlated well with results from end-stage histology, biochemistry and biomechanical testing, which proved that despite the onset of the remodelling phase, tendon healing was not completed after a period of 24 weeks. Randomized controlled long-term studies including naturally occurring tendinopathies are necessary to put the results of the current study into perspective before intralesional AT-MSC injection can be recommended (or rejected) as a viable option for the treatment of tendon disease.

## Additional files

Additional file 1: Table presenting the gradually increasing exercise programme adapted from Bosch et al. [45] with permission. (DOCX 14 kb)

Additional file 2: Semi-quantitative four-point scale according to Aström and Rausing [60], modified by Bosch et al. [45]. (DOCX 15 kb)

## Abbreviations

ADNC: Adipose-derived nucleated cell
AT: Adipose derived
BM: Bone marrow
COMP: Cartilage oligometric matrix protein
CSA: Cross-sectional area
DICOM: Digital Imaging and Communications in Medicine
DMEM: Dulbecco’s Modified Eagle’s Medium
EDTA: Ethylenediaminetetraacetate
FBS: Fetal bovine serum
GAG: Glycosaminoglycan
H&E: Haematoxylin and eosin
HEPES: 4-(2-Hydroxyethyl)-1-piperazineethanesulfonic acid
HLys: Hydroxylysine
HP: Hydroxylysylpyridinoline
Hyp: Hydroxyproline
ICC: Intraclass correlation coefficient
LP: Lysylpyridinoline
Lys: Lysine
MSC: Mesenchymal stromal cell
NaCl: Sodium chloride
SD: Standard deviation
SDFT: Superficial digital flexor tendon
TCSA: Total cross-sectional area
TES: Total echogenicity score
TFAS: Total fibre alignment score
UTC: Ultrasound tissue characterization
