# Supplementary material for: Effect of single intralesional treatment of surgically induced equine superficial digital flexor tendon core lesions with adipose-derived mesenchymal stromal cells: a controlled experimental trial
Source: Stem Cell Res Ther. 2017 Jun 5;8:129. doi: 10.1186/s13287-017-0564-8 (PMC5460527; doi:10.1186/s13287-017-0564-8)
Supplement: Supplementary file 1 — Table presenting the gradually increasing exercise programme adapted from Bosch et al. [45] with permission. (DOCX 14 kb) [file 13287_2017_564_MOESM1_ESM.docx]

**Gradually increasing exercise programme adapted from Bosch et al. 2010 [45]**

| **Week after lesion induction** | **Walking (min / day)** | **Trot (min / day)** |
| --- | --- | --- |
| 1 - 3 | Box rest | Box rest |
| 4 - 6 | 10 | - |
| 7 - 10 | 20 | - |
| 11 - 14 | 30 | - |
| 15 - 18 | 40 | - |
| 19 - 21 | 35 | 5 |
| 22 - 24 | 30 | 10 |

min = minutes
