# Supplementary material for: Effect of single intralesional treatment of surgically induced equine superficial digital flexor tendon core lesions with adipose-derived mesenchymal stromal cells: a controlled experimental trial
Source: Stem Cell Res Ther. 2017 Jun 5;8:129. doi: 10.1186/s13287-017-0564-8 (PMC5460527; doi:10.1186/s13287-017-0564-8)
Supplement: Supplementary file 2 — Semi-quantitative four-point scale according to Aström and Rausing [60], modified by Bosch et al. [45]. (DOCX 15 kb) [file 13287_2017_564_MOESM2_ESM.docx]

**Semi-quantitative four point scale according to Aström and Rausing (1995) [60], modified by Bosch et al. (2010) [45]**

*Scale:*

0 = normal appearance, 1 = slightly abnormal, 2 = moderately abnormal, 3 = markedly abnormal

*Features of sub-scores:*

- Fibre structure (0 = linear, no interruption; 3 = short with early truncation)
- Fibre alignment (0 = regularly ordered, 3 = no pattern identified)
- Morphology of tenocyte nuclei (0 = flat; 3 = round)
- Variations in cell density (0 = uniform, 3 = high regional variation)
- Vascularization (0 = absent; 3 = high)

*Scores:*

- Fibre structure + Fibre alignment = structural integrity
- Morphology of tenocyte nuclei + variations in cell density + vascularization = metabolic activity
